# An Improved Method of Amputating a Cancerous Breast; with an Account of Two Cases in Which It Was Performed with Success

**Published:** 1783

**Authors:** 


IV. *An Improved method of amputating a cancerous breast; with an account of two cases in which it was performed with success. Communicated in a letter to Dr.* Simmons, *by Mr.* Henry Fearon, *Surgeon of the Surry Dispensary.*

NOtwithstanding the general opinion, that cancerous complaints depend on the habit or constitution, and therefore cannot be cured by by any topical treatment or operation, yet as men of the greateſt eminence in the medical profeſſion have hitherto been unſucceſsful in their attempts to diſcover an internal remedy or method of treatment, capable of effecting a radical cure, extirpation by the knife ſeems to be the only reſource left in theſe melancholy caſes; and when performed by a ſkilful ſurgeon it often proves ſucceſsful. For we well know, that although the patient may have a diſpoſition to cancer, yet without the occurrence of an exciting cauſe, the diſeaſe may never again appear. I therefore preſume that an humble attempt to introduce any improvement of an operation, which is ſo often neceſſary to be performed, will be acceptable to the younger part of the profeſſion, and, I flatter myſelf, will be treated with candour by men of eſtabliſhed reputation and ſuperior abilities. My wiſh is to ſee it encouraged and adopted if merit and utility recommend it, but, if deſtitute of theſe, rejected.

Elizabeth Benham of Walworth applied to me to remove her right breaſt. The account ſhe gave of her caſe was, that as long as ſhe could remember ſhe had perceived a ſmall lump in her breaſt, which had gradually increaſed in ſize; and that about ten years before I ſaw her ſhe

ſhe had taken the opinion of Mr. Smith, ſurgeon of St. Thomas's Hoſpital, who adviſed the removal of the diſeaſed part; but that ſhe did not chooſe to ſubmit to an operation at a time when ſhe ſuffered no inconvenience except from its weight, and at times a dull heavy pain. In April 1782 the ſize of the tumour encreaſing, and the pain becoming more acute, ſhe was adviſed to ſee my learned friend and colleague, Dr. Sims, whoſe opinion agreed with that of Mr. Smith. The gland was very large, of an unequal ſurface, very knotty, and felt through its whole ſubſtance perfectly indurated. The veins of the ſkin were varicous, and the nipple was ſhrunk out of ſight. She was forty-eight years old, and of a delicate conſtitution, but her general health was good.

In performing the operation I made an horizontal inciſion a little below the nipple, nearly in the direction of the rib, with a ſtrait diſſecting knife. The inciſion, which was of greater extent than the tumour, occaſioned but little more pain than a ſmall inciſion would have done, and had this great advantage, that it enabled me with facility perfectly to remove the whole of the diſeaſed part. In performing this operation ſeveral arteries bleed very freely, but theſe these need not be regarded, though they are apt to alarm a young operator, who from his embarraſſment may make too much haſte to finiſh the operation. It is neceſſary therefore that we ſhould be guarded againſt every thing of this nature, by conſidering that no danger can ariſe from diſſecting freely, and that if the leaſt part of the diſeaſed maſs be left unremoved, the operation has been performed to no purpoſe *. The edges of the wound were brought into contact, and retained by ſlips of ſticking plaiſter. They united by the firſt intention, and the cicatrix was completely formed in ten days. She was only two days confined to her room, and walked out on the fourth.

The tumour weighed three pounds and ten ounces. On cutting into it, it was extremely hard and diſeaſed through its whole ſubſtance, with ſeveral ſmall cyſts containing a yellow, gelatinous, curdled pus.

Soon after this I was conſulted by Mary Smith, aged forty, who had diſcovered a lump

* An inſtance of this kind occurred lately, where the operation was performed a ſecond time, ſix months after the firſt, as the whole of the diſeaſed part had not been removed by the firſt operation.

 in in her breaſt, about five months before ſhe applied to me. It was perfectly indolent even upon being handled. It had a ſtoney incompreſſible kind of hardneſs. I told her I was of opinion, that it was a true ſchirrus, and accordingly recommended the operation. She left me much diſſatisfied, and I heard no more of her for a fortnight, during which time ſhe had often been to an eminent ſurgeon, and then returned to ſubmit to the operation. The diſeaſe in the above ſhort time had increaſed very rapidly. The ſkin was become ſmooth and inflamed, and appeared as if it would ſhortly break out into a cancerous ſore. There were little lumps round the breaſt, and a gland enlarged in the axilla to the ſize of a cheſnut, which looked as if abſorption had taken place. I now told her, that from the rapid increaſe and unfavourable appearance of the diſeaſe, her chance of a cure from the operation would be extremely uncertain. Her reply was, that ſhe ſuffered ſuch acute and almoſt conſtant pain, that ſhe would run any riſk. I operated in the ſame manner as in the former caſe, removing all the indurated glands. That in the axilla was deeper ſeated than it had appeared to be from the external feel. I likewiſe removed all that part of the ſkin, which which was either difcoloured or adhered to the difeafed mafs. Still there was enough left to admit of a perfect approximation of the edges of the wound, which healed by the firft intention. On cutting into the tumour after it was removed, there appeared two cyfts, one of which contained about three ounces of ferum, and the other a curdled matter tinged with blood. Ulceration had taken place in the infide.

From thefe cafes I think the following inferences may be drawn: viz. That when a fchirrous breaft is moveable, and does not adhere either to the fkin above, or below to the adipofe membrane, mufcles, &c. the making a fimple incifion fo as to diffect out the breaft will be fufficient, and an eafy remedy when fubmitted to in time, as this method requires fo little confinement: 2dly, That as foon as the difeafe is clearly afcertained it ought to be removed; for though a fimple fchirrus may remain indolent and inoffenfive for a great many years without producing its bad confequences (and hence give room for the boafted fpecifics receiving the undeferved credit of having prevented the farther progrefs of the difeafe) yet it may as fuddenly and rapidly prove deftructive, if not attended to by a careful obferver: 3dly, That frequent pain, increafe of fize, enlarged lumps round the breaft, and a fchirrous gland in the axilla ought not to difcourage us from performing the operation.

I have found the above method equally fuccefsful after the removal of a fchirrous tefticle, as by bringing the edges of the wound into contact, they will heal by the firft intention. Therefore, with all due deference to men of the greateft abilities who ftill perfevere in the ufual mode of filling the void fpace with dry lint, I venture to affert, that this is unneceffary. If facts and experience fupport me in this opinion, I hope no one will be fo illiberal as to impute it either to vanity or the affectation of fingularity.

